# Analytical Assessment of the Vela Diagnostics NGS Assay for HIV Genotyping and Resistance Testing: The Apulian Experience

**DOI:** 10.3390/ijms23052727

**Published:** 2022-03-01

**Authors:** Maria Addolorata Bonifacio, Chiara Genchi, Antonella Lagioia, Vincenza Talamo, Anna Volpe, Maria Addolorata Mariggiò

**Affiliations:** 1 Section of Experimental and Clinical Pathology, Department of Biomedical Sciences and Human Oncology, University of Bari Aldo Moro, 70124 Bari, Italy; maria.bonifacio@uniba.it (M.A.B.); chiaragenchi.policlinico@gmail.com (C.G.); vincenzatalamo.policlinico@gmail.com (V.T.); 2 Clinic of Infectious Diseases, Department of Biomedical Sciences and Human Oncology, University of Bari Aldo Moro, 70124 Bari, Italy; antonella.lagioia@uniba.it

**Keywords:** next-generation sequencing (NGS), human immunodeficiency virus (HIV), genotyping, Vela Diagnostics, Sanger sequencing (SS), resistance-associated mutations (RAM), protease inhibitors (PIs), integrase strand transfer inhibitors (INSTIs), nucleoside reverse transcriptase inhibitors (NRTIs), non-nucleoside reverse transcriptase inhibitors (NNRTIs)

## Abstract

Drug-resistance monitoring is one of the hardest challenges in HIV management. Next-generation sequencing (NGS) technologies speed up the detection of drug resistance, allowing the adjustment of antiretroviral therapy and enhancing the quality of life of people living with HIV. Recently, the NGS Sentosa^®^ SQ HIV Genotyping Assay (Vela Diagnostics) received approval for in vitro diagnostics use. This work is the first Italian evaluation of the performance of the Vela Diagnostics NGS platform, assessed with 420 HIV-1 clinical samples. A comparison with Sanger sequencing performance is also reported, highlighting the advantages and disadvantages of the Sentosa^®^ NGS assay. The precision of the technology was studied with reference specimens, while intra- and inter-assay reproducibility were evaluated for selected clinical samples. Vela Diagnostics’ NGS assay reached an 87% success rate through 30 runs of analysis in a real-world clinical context. The concordance with Sanger sequencing outcomes was equal to 97.2%. Several detected mismatches were due to NGS’s superior sensitivity to low-frequency variants. A high accuracy was observed in testing reference samples. Repeatability and reproducibility assays highlighted the good performance of the NGS platform. Beyond a few technical issues that call for further optimization, the key improvement will be a better balance between costs and processing speed. Once these issues have been solved, the Sentosa^®^ SQ HIV Genotyping Assay will be the way forward for HIV resistance testing.

## 1. Introduction

More than 40 million people all over the world are currently living with HIV, the retrovirus responsible for the HIV/AIDS pandemic [1]. After almost 40 years from the isolation of HIV, this retrovirus is still a world health threat. Indeed, in 2020, HIV claimed the life of 680,000 people [2].

Combined antiretroviral therapy (cART) suppresses HIV replication, preventing the development of AIDS syndrome and replacing it with a manageable chronic disease [3]. However, a cure with which to eradicate HIV is currently unavailable, partly because of the intrinsic genetic variability of this infectious agent [4]. cART triggers the emergence of HIV-resistant variants, selected under drug pressure [5]. Several studies have explored the impact of low-frequency resistance-associated mutations (RAMs) on virological failure [6,7]. Nucleosidic and non-nucleosidic reverse transcriptase inhibitors (NRTIs and NNRTIs, respectively), as well as protease inhibitors (PIs), have resulted in drug classes particularly affected by RAMs [8]. Therefore, WHO and the International Antiviral Society (IAS-USA) guidelines have stated that resistance testing is a cornerstone of cART success and should be performed immediately before treatment and after its failure [9,10].

Dideoxynucleoside Sanger sequencing, the traditional approach exploited in clinical settings, as well as the most innovative next-generation sequencing systems, enable RAMs detection and provide genotyping information useful to the clinician [11]. However, NGS approaches will likely replace the traditional Sanger sequencing because of high-throughput capacity, enhanced sensitivity, shorter turn-around time and cost-effectiveness [12]. In particular, deep sequencing NGS strategies are characterized by excellent sensitivity, essential to search for low-frequency variants. In addition, NGS approaches allow for mixture deconvolution, which is particularly useful in the study of HIV [13]. However, most of the NGS platforms on the market (i.e., Illumina, Roche) are certified for research use only and thus are still not suitable for clinical diagnostic applications. In 2019, the Vela Diagnostics NGS platform achieved FDA approval for HIV genotyping and resistance testing for in vitro diagnostic purposes [14]. The Vela Diagnostics NGS system is nowadays the only semi-automated NGS assay approved by the FDA, EMA, TGA and HAS for in vitro diagnostic use on people living with HIV.

A few independent studies have described the performance of the Vela Diagnostics NGS platform in a research context, often with a small number of selected patients [15,16]. In this study, which is the first performed in Italy, the evaluation of the Vela Diagnostics system is reported in terms of the success rate, accuracy, repeatability and reproducibility of the results. The analytical assessment of the NGS platform has been accomplished in a real-world clinical setting, shedding light on the strengths and drawbacks of the NGS system.

Compared to Sanger sequencing outcomes, the NGS technology provided reliable results, even if there is still room for its improvement.

## 2. Results

### 2.1. NGS Analyses Performed on Clinical Samples

The Sentosa^®^ SQ HIV Genotyping Assay was performed to check for RAMs upon treatment failure or before the start of cART. Table 1 summarizes the characteristics of the PLWH (*n* = 420) in a follow-up at the Policlinico Hospital, Bari, tested with the Vela Diagnostics NGS system.

**Figure 1:**
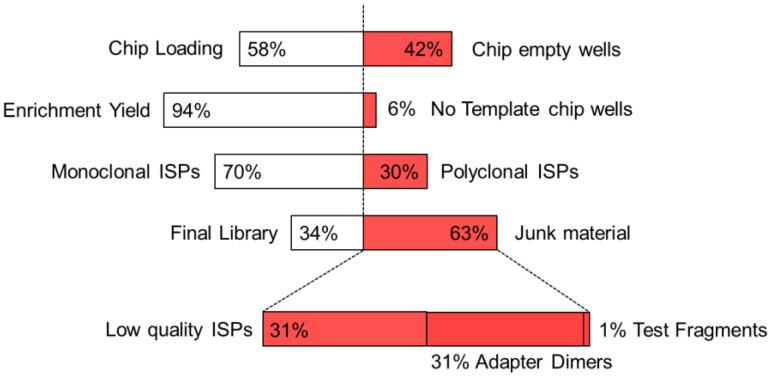
Critical phases impacting the Vela Diagnostics NGS run success rate. From top to bottom: chip loading percentage (filled vs. empty wells), template enrichment percentage (enriched vs. no template wells), percentage of Clonal Ion Sphere™ particles (ISPs) (monoclonal vs. polyclonal ISPs) and final library percentage (percentage of valid reads vs. junk material, subdivided in low quality DNA, adapter dimers and test fragments).

A total of 30 runs were completed on the Sentosa^®^ NGS platform, with a success rate of 87% (4/30 runs failed to meet the quality control acceptance criteria and required reanalysis). Figure 1 depicts the main parameters affecting sequencing success, i.e., chip loading percentage (filled/empty wells on the Sentosa^®^ SQ 318 chip), template enrichment, clonality of Ion Sphere™ particles (ISPs) and final library percentage. With this instrument version of the Sentosa^®^ SQ HIV Genotyping Assay, the chip loading phase was performed manually for each of 30 runs, leading to a median chip loading percentage of 58.4% (IQR 50.8–62.6%). Even if this step was performed by two skilled operators, it proved to be an unavoidable source of variability. Nevertheless, though better performance could be achieved, the chip loading step never determined the run failure. Furthermore, the template enrichment phase was generally successful (median 94.0%, IQR 91.3–97.0%), as confirmed by the high number of monoclonal ISPs (median 70.0%, IQR 66.0–72.8%). On the other hand, the library preparation was the most critical step, mainly affected by the presence of low-quality ISPs (median 31.5%, IQR 26.0–37.5%) and by the number of adapter dimers (median 30.5%, IQR 24.3–37.5%).

The latter, i.e., amplicons with inserts shorter than 8 bp, could be significantly reduced through a manual clean-up protocol, already automated on the updated version of the Sentosa^®^ SQ HIV Genotyping Assay. Beyond the complexity of the Sentosa^®^ NGS platform, it is worth noting that 302/420 samples were successfully analyzed, while 34 samples (8.1%) led to low-coverage warning messages. No amplicons were reported for 82/420 samples (19.5%). The median coverage of the PRO/RT region was 3701 (IQR 1866–5858), while that of the INT region was 5292 (IQR 3143–8694).

HIV-1 subtype distribution was B 65.3% (*n* = 267), CRF02_AG 10.8% (*n* = 44), A1 9.0% (*n* = 37), G 7.6% (*n* = 31), C 6.6% (*n* = 27), F1 2.9% (*n* = 12), D 1.5% (*n* = 6), CRF01_AE 1.5% (*n* = 6). Mirroring the most up to date Apulian data on HIV infection monitoring, the PLWH analyzed in this study were mainly B subtype young men, with a median age of 44, having been infected with HIV-1 by the sexual route (MSM) [17]. In addition, a group of foreign heterosexual people, mainly from Africa, was also well represented, reflecting the migratory fluxes crossing Apulia in recent years [18]. When the Sentosa^®^ Assay was performed, 17% of patients had less than 300 cp/mL of HIV plasmatic RNA and a median of CD4 cells/µL equal to 700 (454–896). Conversely, 20% of the tested PLWH had a CD4 cell count ≤200 cells/µL and a median viral load equal to 151,224 cp/mL (IQR 69,714–360,330 cp/mL).

**Figure 2:**
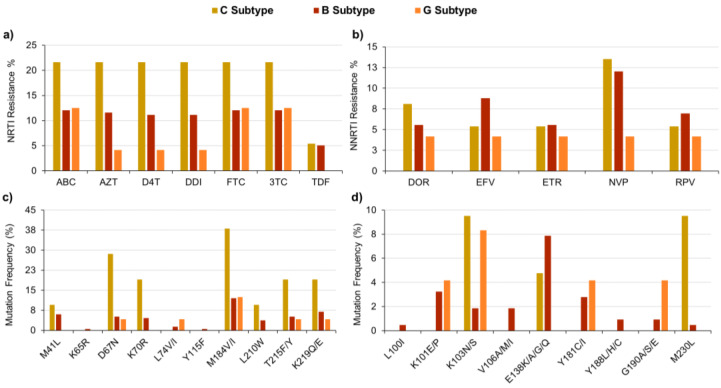
Percentage prevalence of resistance for NRTI (**a**) or NNRTI (**b**) drugs. Occurrence of RAMs related to NRTI (**c**) and NNRTI (**d**) drug-resistance development.

The Sentosa^®^ SQ HIV Genotyping Assay highlighted the presence of several RAMs, mainly targeting reverse transcriptase but also the protease and integrase enzymes. Figure 2a details the percentages of samples resistant to seven common NRTI drugs, showing that they often belonged to C, B or G subtypes. The most frequent RAMs reported by the Sentosa^®^ assay are described in Figure 2c. Even if in this study the sample number/subtype ratio was not the same for each group, overall, the results were in agreement with the mutation prevalence data reported in HIVdb [19]. Indeed, the occurrence of the M184V/I mutation within the tested samples was less frequent in B/G subtype than in C subtype samples, leading to reduced susceptibility to ABC, 3TC and FTC [20]. In addition, the Type 1 (i.e., M41L, L210W and T215Y/F) and Type 2 (i.e., D67N, K70R, T215Y/F and K219Q/E) patterns of thymidine-analog mutations (TAMs) were observed in 9.5% and 14.3% of C subtype samples, respectively. The same patterns were also detected, although less frequently, in B subtype samples (4.6% and 5.1%). The TAM patterns, mainly Type 1 also combined with Type 2, played a pivotal role in the development of resistance to ABC, AZT, D4T, DDI and TDF drugs [21].

Concerning NNRTIs (Figure 2b,d), C and B subtypes developed RAMs with similar frequencies, while G subtype samples seemed to be generally more susceptible to this drug class. The K103N/S was the most common RAM, often detected in C and G subtype samples, according to findings reported in the literature [22,23]. This non-polymorphic mutation negatively affected the susceptibility to NNRTIs, mainly NVP and EFV [24]. Moreover, the M230L mutation occurred in 9.5% of C subtype patients, often combined with the accessory mutation A98G, decreasing the effectiveness of DOR, EFV, NVP and RPV [25]. On the other hand, ETR resistance was mainly driven by E138K/A/G/Q and Y181C/I mutations [26,27].

Furthermore, the prevalence of RAMs affecting HIV-1 protease was lower than 10% for all the studied subtypes. Similarly, it was ≤5% for RAMs targeting the integrase enzyme. Indeed, drugs belonging to PI and INSTI classes were still effective on more than 95% of the tested patients.

### 2.2. Comparison with Sanger Sequencing

**Figure 3:**
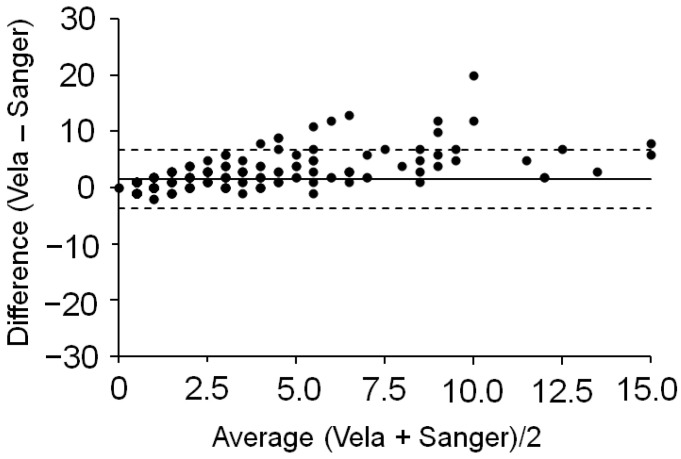
Bland–Altman plot. Dotted lines indicate the 95% limit of agreement of the two sequencing methods, while the solid line represents the bias (i.e., the average difference between Sentosa^®^ and Sanger sequencing platform performances).

To compare the outcomes of the Sentosa^®^ SQ HIV Genotyping Assay with those of direct Sanger sequencing, 23 samples were tested with both technologies. The limited plasma volumes available did not allow the performance of both sequencing methods on a larger subset of patients. The sample subtype was mainly B (91%), while the median viral load was equal to 21,951 cp/mL (IQR 2010–59,729). All samples passed quality controls on the Sentosa^®^ NGS platform, even if 6/23 samples had viral loads <1000 cp/mL, not recommended by Vela Diagnostics. Nevertheless, they were included because such low viral loads are frequently observed in clinical settings. Viral subtype assessment achieved a 100% matching for the two sequencing approaches. Concerning variants detection, Sanger sequencing and the Sentosa^®^ SQ HIV Genotyping Assay showed a concordance of 97.2%, in agreement with previous literature findings [28]. The main differences were due to low-frequency variants, detected by the Sentosa^®^ NGS platform but not by Sanger sequencing. Therefore, the Bland–Altman plot in Figure 3 showed most of the measurements (~81%) above zero and a positive bias (d = 1.5) in favour of the Sentosa^®^ NGS system.

Nevertheless, both methods were effective in RAMs detection, providing the same drug resistance interpretation for 21/23 patients. Indeed, the obtained sequences were studied through the HIVdb v.9.0 genotyping resistance program, assigning penalty scores to each mutation and calculating a resistance level (from 1 to 5) for each of the most common available drugs [29]. It was observed that two RAMs (E138A, M230I) significantly affected the susceptibility to NNRTI drugs, and one RAM (E138K) impaired the response to INSTIs (Table 2). These mismatches occurred in two different samples belonging to the B subtype. Conversely, no RAMs mismatches were observed within the protease sequences. In just one case, the accessory mutation L10LFIV within the protease region was absent in the Sentosa^®^ report, but it was found by loading manually the relevant FASTA sequence on the HIVdb program [29]. In addition, potential G-to-A hypermutations associated with APOBEC signatures were studied on NGS-derived and Sanger-derived sequences by the HIVdb program. Concerning NGS samples, 3/23 had three or more APOBEC mutations each, while SS reported just one APOBEC mutation for the same 3/23 samples. Beyond the findings related to these samples, for which unusual mutations were also observed, the NGS technology was generally more prone to detect APOBEC signatures than Sanger sequencing. This result is reasonable, since NGS highlights much better than Sanger sequencing the PCR artifacts caused by low viral loads and/or improper RNA extraction. The observed differences were statistically significant for PRO/RT regions (*t*-test, *p* < 0.05) but not for INT sequences, in agreement with literature findings [30].

### 2.3. NGS Sequencing of Reference Samples

Reference samples (QCMD, Glasgow, Scotland), with known HIV-1 subtype and set of variants, were analyzed by the Sentosa^®^ SQ HIV Genotyping Assay (Table 3 and Table 4), resulting in 100% correct subtype assignment and 99.35% detection of the overall variants (*n* = 461/464). The median coverage of both PRO/RT and INT regions was variable but always higher than 1000, i.e., the minimum value recommended by Vela Diagnostics. For all the reference samples, the throughput was ≥2,500,000 bp and the reads mapping process was successful for more than 99% of reads. Completeness was always higher than 94% and the median amplicon length was 146 ± 16 bp for PRO/RT and 153 ± 14 bp for the INT region.

#### 2.3.1. Accuracy Performance of the Vela Diagnostics NGS Platform

Reference samples 1, 2 and 5 obtained a 100% match with expected variants. The RT region had 100% homology with the known sequence for all the samples, while a mismatch occurred within the protease region belonging to Sample 4. Indeed, the A71AT mixture (RCT triplet) was reported instead of the wildtype codon (T71, GCT triplet) due to the unambiguous attribution of the first triplet nucleotide by the Sentosa^®^ NGS platform. Sequencing was performed in duplicate for all the samples and the unique discordant codon (A71T, protease region) resulted in a mutation frequency of 16.2 ± 1.4% (Table 3). Moreover, integrase sequences were 100% identical to those expected for samples 1, 2 and 5. Conversely, one mismatch was detected in Sample 4, i.e., T125A. Instead of the expected mixture of ACG and GCG triplets (T125TA), the Sentosa^®^ SQ HIV Genotyping Assay reported only a GCR triplet in one sample and a GCG triplet in its duplicate, leading in both cases to alanine calling (Table 4). Furthermore, for Sample 3, the Sentosa^®^ SQ HIV Genotyping Assay reported an AYG codon, instead of the expected ACG codon, thus leading to an ambiguous threonine/methionine attribution (Table 4).

#### 2.3.2. Intra-Assay Repeatability

**Figure 4:**
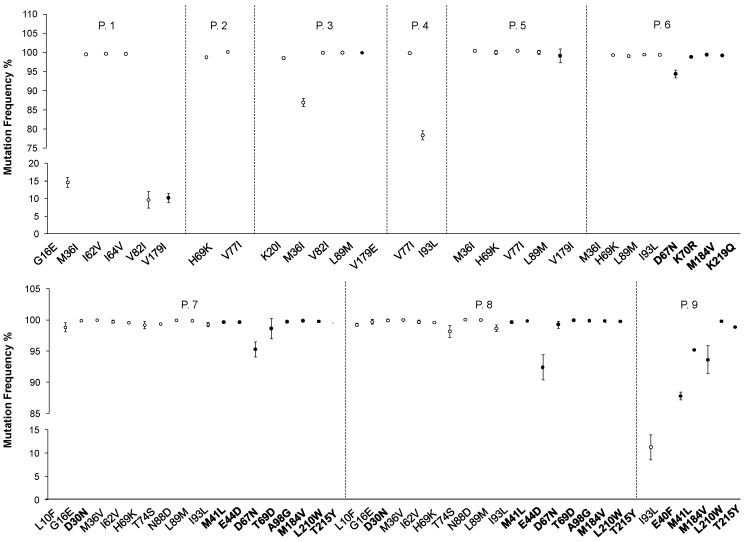
Frequency (mean ± standard deviation) of variants and RAMs (in bold) detected by the Sentosa^®^ SQ Reporter among the nine patients studied in the intra-assay reproducibility test. White dots indicate variants/RAMs occurring in the protease region, while black dots indicate reverse transcriptase variants/RAMs.

Nine clinical samples were tested twice in a run to evaluate intra-assay repeatability performance (Table 5). The coverage of PRO/RT and INT regions had a median (IQR) equal to 5160 (3374–7973) and 3718 (2298–4800), respectively. A total of 27 RAMs were reported, in addition to 43 target variants detected by the Sentosa^®^ SQ HIV Genotyping Assay, mainly in the protease region (Figure 4, white dots), with a median of 5 ± 4 variants per patient.

Of the detected variants, 9% (*n* = 4) had a frequency below 20%. The viral load requirements suggested by the supplier (HIV-1 RNA > 1000 cp/mL) were fulfilled for 8/9 samples, while P. 5 had a viral load of 737 cp/mL. However, all samples passed quality control criteria and no warning messages were reported by the Sentosa^®^ NGS platform. Comparing the two sequence sets obtained for each patient, it is worth noting that seven out of nine samples provided overlapping results, with no mismatches in variants detection. Nevertheless, the protease region of sample P. 2_B was only partially sequenced (codons 1–66), thus the H69K and V77I variants occurring in the unsequenced region were not detected. Similarly, for sample P. 5_B, the first 60 codons of the protease region were not sequenced, leading to the M36I variant being missing. With these two exceptions, complete sequences were obtained for all the other samples. The variant frequencies were superimposable (median frequency difference of 0.09%, IQR 0.84–2.29%) both for those occurring in the protease and reverse transcriptase regions (Figure 4). The drug-resistance interpretation reports generated by the Sentosa^®^ SQ Reporter server did not highlight the presence of variants/RAMs within the integrase regions of these nine patients. However, the patients’ FASTA consensus sequences, manually loaded on HIVdb v.9.0, led to the detection of an additional accessory mutation (D232N) causing potential low-level resistance to RAL and EVG in P. 9, not highlighted by the automatic analyses performed by the Sentosa^®^ SQ Reporter. Additional variants (Table S1, Supplementary Materials) were detected within integrase (*n* = 146) as well as protease (*n* = 41) and reverse transcriptase (*n* = 224) regions. Nevertheless, none of the observed additional variants (*n* = 411) had an impact on drug susceptibility.

#### 2.3.3. Inter-Assay Reproducibility

Table 6 describes the results relevant to fourteen patients (*n* = 28) whose plasma samples were studied in two different runs to assess Sentosa^®^ SQ HIV Genotyping inter-assay repeatability. Nine patients had a viral load >1000 cp/mL, as recommended by Vela Diagnostics. For PRO/RT and INT regions, the coverage was variable, with a median (IQR) of 1093 (643–7154) and 925 (265–6923), respectively. Overall, 8 RAMs were identified (1 accessory mutation and 2 major mutations in the INT region, 5 mutations in the RT region) and 80 variants were found (median 6 ± 4 variants per patient), most often in the protease region. Only 4 variants occurred with <20% frequency. For seven patients, warning messages were reported (Table 6, sample names with asterisks) because of low throughput (still ≥125,000 bp) and, in four cases, partial sequences were obtained. The integrase region was not sequenced in P. 19_Run A, while the PRO/RT was missing in P. 19_Run B, not allowing comparison of the detected variants for this patient. Nevertheless, no mismatches were detected for nine patients. A total of five mismatches were found, three of which were relevant to the H69 codon in the protease region (H69K/Q). No mismatches were related to unsequenced regions. A reliable concordance in inter-assay variant frequencies was observed for each region, with a median (IQR) frequency difference equal to 0.54% (IQR 0.16–1.81%). In addition, no statistically significant differences were found comparing intra- and inter-assay performances in variant frequency percentages (*t*-test, *p* > 0.05). Additional details are reported in Table S2, Supplementary Materials.

## 3. Discussion

This study reports the analytical assessment of the Sentosa^®^ SQ HIV Genotyping Assay in a real-world clinical context. For the first time, 420 Italian people living with HIV were tested with the Vela Diagnostics NGS platform. For 30 total runs, the success rate was equal to 87%. The result herein described was achieved by two well-trained operators, with years of experience using the Vela Diagnostics NGS platform and specializing in molecular biology techniques. Previous studies of the same NGS system reported a success rate of 56% [31]. The same platform, exploited to study HIV-1 DNA extracted from buffy coats, achieved a 72.5% success rate [15]. Moreover, Bocket and co-workers reported that the success rate was around 60% when manually extracted HIV-1 DNA samples were added into the Vela Diagnostics NGS workflow [32].

Several factors, independent of the sequencing technology, have a significant impact on the overall sequencing success rate. Indeed, the viral load, the extraction protocol, the starting material (i.e., DNA extracted from whole blood/RNA extracted from plasma samples) and amplicon length are all tunable parameters to optimize the sequencing success rate. In this respect, Lapointe et al. focused on the effect of HIV plasma viral loads on the success rate of Sanger sequencing and Illumina MiSeq technologies [33]. In their work, they reported that both platforms reached the 80% success rate on a large dataset consisting of more than 800 samples. Nevertheless, for samples with viral loads in the range of 300–1000 copies/mL, a success rate below 60% was achieved by Sanger sequencing. The outcome further dropped to 40% for viral loads <1000 cp/mL if amplification was not reattempted through a backup protocol [33]. Moreover, Sanger sequencing reached similar results (45% success rate) for DNA extracted samples, as reported by Raymond and co-workers [16]. Therefore, rather than the type of starting material, viral load seems to drive the sequencing success rate. Indeed, also on the Vela Diagnostics NGS platform, beyond quality control issues, the encountered failures generally occurred with low viral load samples (~100 cp/mL), even if the limit of detection of the Sentosa^®^ SQ HIV Genotyping Assay herein experienced was far below that stated by Vela Diagnostics (1000 cp/mL). Indeed, according to a previous study [34], our results reported proper sequence coverage, correct subtyping and successful detection of RAMs for samples with a viremia equal to 300 cp/mL.

On the other hand, no correlation was observed between sample failure and HIV-1 subtype. Indeed, the non-B subtype sequences were generally successfully amplified by the NGS platform, and their analyses led to effective RAMs detection.

This finding was confirmed by the high levels of accuracy reached for reference samples of all subtypes. No correlation between viral load and number of mismatches or sequence completeness was detected. Furthermore, intra-assay repeatability and inter-assay reproducibility performances were also in agreement with those claimed by Vela Diagnostics (100% reproducibility, 98.5% clinical sensitivity and 99.82% variant detection correctness).

Comparing NGS and Sanger sequencing systems, the results agreement reached 97.2%. Although the patient subset herein studied only included 23 samples, the obtained data were in good agreement with previous reports [16,28]. Despite the low number of studied samples, it can be observed that most of the mismatches herein reported were due to low frequency variants (~20%) missed by Sanger sequencing. Nevertheless, considering the different chemical sequencing mechanisms on which the two technologies are based, a 2.8% bias seems reasonable.

Furthermore, a previous study [28] compared the Vela Diagnostics system to other NGS technologies, certified for research-use-only (i.e., Illumina MiSeq and 454 GS-FLX Roche), establishing that the Vela NGS platform missed minor variants below 20% frequency. Nevertheless, the 20% variant frequency is generally accepted as a clinically relevant threshold, thus the obtained outcome is suitable for in vitro diagnostic use. In addition, Vela Diagnostics claimed the effective detection of 5%-frequent variants for samples with a viral load >4000 cp/mL, similarly to other NGS technologies exploited for research purposes [35]. However, such high viral loads often occur only before cART starting and are rarely detected during therapy and within a regular follow-up regimen. Therefore, the high sensitivity of the Vela Diagnostics NGS platform is helpful for first diagnoses and for molecular epidemiology but is currently less useful in common clinical practice. This point highlights the high potential of the described NGS system, which seems particularly suitable for large medical centers rather than local, unspecialized laboratories. Nevertheless, the platform marketed by Vela Diagnostics aims at major automation of several hands-on steps and data mining procedures to provide HIV genotyping and resistance testing in low- and middle-income countries. The automation level of HIV RNA extraction, amplification and template library building is still growing, such that the emulsion PCR step of the original assay has been now replaced by a more efficient isothermal PCR phase. Furthermore, the manual chip loading step has been integrated into a robotic liquid handling instrument which dispenses PCR reagents too. In addition, a camera was also mounted on the new Sentosa^®^ SX101 instrument. All these adjustments aim at reducing variability, which is otherwise difficult to control in a complex workflow, involving several hands-on steps and instrumental tasks. Thus, the achievement of a major level of automation will play a key role in the diffusion of the Vela Diagnostics NGS platform in local laboratories. Nowadays, this system is still the only semi-automated NGS assay certified for in vitro diagnostic use in several countries (i.e., USA, Europe, Australia, Thailand and Japan).

Another major factor in the in vitro diagnostic NGS market will always be cost. Indeed, to be truly cost-effective, the Sentosa^®^ SQ HIV Genotyping Assay should be performed in specialized diagnostic centers ensuring a proper number of patients asking for testing. Otherwise, it is difficult to provide useful results and carry out runs of 15 samples each without delaying turn-around time. It should be mentioned that, in order to overcome this issue, Vela Diagnostics has recently marketed a versatile solution, the ViroKey™ SQ FLEX Genotyping Assay, which combines HIV-1, CMV, HBV and HCV testing in a single NGS run, enabling local laboratories with a limited number of patients to afford the instruments and reagent costs.

Furthermore, it is essential to optimize and standardize data mining and automatic report operations to achieve meaningful clinical conclusions. Indeed, algorithm updates (i.e., the latest version of HIVdb) should be implemented on all the Sentosa^®^ SQ Reporter servers, otherwise there will be the risk of bias between the results of analyses based on older databases versions and those based on the most up to date knowledge of HIV drug resistance. This was the case with an accessory mutation (D232N) causing potential low-level resistance to RAL and EVG that was only detected by manual loading of a patient’s integrase sequence on HIVdb v.9.0 (see paragraph 2.3.2). Nevertheless, for all the other samples studied in this work, no relevant differences were observed across the drug susceptibility reports generated by the Sentosa^®^ SQ Reporter server embedding HIVdb v.8.8 and the results manually obtained through HIVdb v.9.0.

The urgent need for a standardized bioinformatic pipeline for HIV genotyping resistance data analysis was stated at the second Winnipeg conference [36] and should be a priority also for Vela Diagnostics, whose platform was designed to be user-friendly and standardized. However, these advantages should not lead to the idea that the obtained automatic reports could be given as they are to clinicians, without the careful interpretation of a skilled operator. Indeed, automated reporting carries the risk of drawing completely misleading conclusions from technical artifacts. Therefore, it is strongly recommended to carefully inspect low-frequency variants (less than 5%), as well as those associated with APOBEC mutations, to distinguish between PCR artifacts and actual mutations. The treatment history of the patient should always be used to endorse drug resistance reasonably emerging under treatment pressure.

The evaluated Sentosa^®^ SQ HIV Genotyping Assay has the advantages of reducing hands-on time and infectious risks for the operators, while providing useful clinical results. With further essential optimizations, the Vela Diagnostics NGS system will make a substantial contribution in the world fight against the spread of HIV drug resistance.

## 4. Materials and Methods

### 4.1. Sample Collection and Data Analysis

Genotyping and drug resistance testing was performed with the Sentosa^®^ SQ HIV Genotyping Assay (Vela Diagnostics, Hamburg, Germany) on 420 patients, on follow-up at the Policlinico Hospital, Bari. Blood samples were collected in EDTA tubes (BD Vacutainer^®^, Milan, Italy); plasma fractions were separated with a Cobas p630 pre-analytical instrument (Roche Diagnostics S.p.A., Monza, Italy) and stored at −80 °C until testing. Before genotyping, routine viral load assessment was performed for each plasma sample by the dual-target COBAS^®^ AmpliPrep/COBAS^®^ TaqMan^®^ HIV-1 Test (v.2.0, Roche Diagnostics S.p.A., Monza, Italy), which provided HIV-1 RNA quantitation over the range of 20–10,000,000 cp/mL (corresponding to 34–17,000,000 IU/mL). The NGS and Sanger techniques exploited in this work are detailed in the following paragraphs.

Reference samples (QCMD, Glasgow, Scotland) were studied to evaluate the precision performance of the Sentosa^®^ SQ HIV Genotyping Assay. Furthermore, intra-assay repeatability was assessed with the patients’ samples (*n* = 5), analyzed in duplicate within the same genotyping run. Inter-assay reproducibility was also monitored, testing plasma samples (*n* = 14) in different runs.

All the presented parameters were reported as percentages, medians (IQR) or means ± standard deviation. Graphs were drawn with GraphPad Prism software v.9.3.0 (San Diego, CA, USA).

### 4.2. Next-Generation Sequencing with the Vela Diagnostics Platform

The Sentosa^®^ SQ HIV Genotyping Assay involves seven main steps. Firstly, RNA extraction from plasma samples was performed with the Sentosa^®^ SX101 instrument (Vela Diagnostics, Hamburg, Germany), followed by an off-board RT-PCR step with a Veriti^®^ Dx 96-Well Thermal Cycler (Thermo Fisher Scientific, Waltham, MA, USA). Then, the Sentosa^®^ SX101 instrument built a pooled library, which underwent a template preparation step on the Sentosa^®^ ST401 system (Vela Diagnostics, Hamburg, Germany). After an enrichment procedure, performed with the Sentosa^®^ ST401e instrument (Vela Diagnostics, Hamburg, Germany), the sequencing step took place on the Sentosa^®^ SQ301 (Vela Diagnostics, Hamburg, Germany), which exploits the Ion Torrent™ system (Thermo Fisher Scientific, Waltham, MA, USA) to perform ultra-deep sequencing. Finally, Sentosa^®^ SQ Suite and SQ Reporter software packages (Vela Diagnostics, Hamburg, Germany) were used for primary and secondary data analysis, respectively. An extraction control (EC) was exploited to monitor RNA extraction and library preparation steps, while a system control (SC) was analyzed as a sample and provided a check for the entire analysis run. The quality of SC assembly, i.e., its run throughput (≥40,000,000 bp), median coverage (≥200), completeness (≥95%) and error rate (<1%), are essential to validate sample results. Indeed, a warning status was reported if these parameters fell under their minimum thresholds. The entire workflow generated FASTA files with consensus sequences relevant to the INT region (codons 1–288) and the PRO region (codons 1–99), concatenated with the RT region (codons 1–386). Nucleotide variants ≥5% were reported using the IUPAC-IUB ambiguity system [37]. Beyond raw FASTA files, the assessed subtype based on PRO and RT sequences was provided within a drug susceptibility report. The latter was automatically generated using Sentosa^®^ SQ Reporter software, exploiting Stanford HIVdb v.8.8, ANRS 29 (2018/11/01) and Rega algorithms (v.10.0.0).

### 4.3. Dideoxynucleoside Sanger Sequencing

HIV RNA was purified from plasma samples using a QIAamp DSP Virus Kit (Qiagen, Milan, Italy). The whole protease region and RT codons 1–335 were reverse-transcribed and amplified using a ViroSeq™ HIV-1 genotyping kit (Celera diagnostics, Alameda, CA, USA) as recommended by the supplier. The integrase region was reverse-transcribed and amplified following an in-house approach reported by Santoro and co-authors [38]. The PCR products were then sequenced on an ABI 3130 genetic analyzer (Applied Biosystems, Carlsbad, CA, USA). The SeqScape™ software (Applied Biosystems, Carlsbad, CA, USA) was exploited for amplicon analyses, reporting the presence of mutations detected by comparison with the HXB-2 (K03455) reference sequence. The DNASTAR package (Lasergene Molecular Biology v. 17, Madison, WI, USA) was also used to assess nucleotide mixtures for positions with a second electrophoretic peak >25%. FASTA sequences were exported and analyzed by the Quality Control Tool available on the Los Alamos web database (http://www.hiv.lanl.gov/content/sequence/QC/index.html, accessed on 10 January 2022).

## Figures and Tables

**Table 1:** Patient characteristics.

Parameter	Median (IQR)	Percentage
Sex (male %)		71.4
Age (years)	45 (35–53)	
HIV-1 viral load (cp/mL)	17744 (327–97859)	
HIV-1 subtype (B %)		65.3
CD45 cell count (cells/µL)	1960 (1336–2471)	
CD4 cell count (cells/µL)	435 (218–698)	
CD4/CD8 ratio	0.46 (0.22–0.73)	

**Table 2:** Resistance levels for NRTI, NNRTI and INSTI drugs, assigned by the HIVdb program to sequences obtained by Sanger sequencing and the Sentosa^®^ SQ HIV Genotyping Assay.

	**NRTI**						
	**ABC**	**AZT**	**D4T**	**DDI**	**FTC**	**3TC**	**TDF**
SS	1	1	1	1	1	1	1
NGS	3	3	1	3	5	5	1
	**NNRTI**						
		**DOR**	**EFV**	**ETR**	**NVP**	**RPV**	
SS		1	1	1	1	1	
NGS		3	3	3	4	4	
	**INSTI**						
		**BIC**	**CAB**	**DTG**	**EVG**	**RAL**	
SS		1	1	1	1	1	
NGS		2	2	2	3	3	

SS: Sanger Sequencing; NGS: Vela Diagnostics next-generation sequencing; ABC: Abacavir; AZT: Zidovudine; D4T: Stavudine; DDI: Didanosine; FTC: Emtricitabine; 3TC: Lamivudine; TDF: Tenofovir; DOR: Doravirine; EFV: Efavirenz; ETR: Etravirine; NVP: Nevirapine; RPV: Rilpivirine; BIC: Bictegravir; CAB: Cabotegravir; DTG: Dolutegravir; EVG: Elvitegravir; RAL: Raltegravir. 1: Susceptible; 2: Potential low-level resistance; 3: Low-level resistance; 4: Intermediate resistance; 5: High-level resistance.

**Table 3:** Assessment of Vela NGS platform performances on EQA samples—PRO/RT Regions.

PRO/RT	Sample 1	Sample 2	Sample 3	Sample 4	Sample 5
Subtype	CRF_02AG	C	B	D	C
Median coverage	10997	3134	6771	9912	8694
Total Mapped Reads	166413 (99.43%)	39642 (99.14%)	124209 (99.69%)	174423 (99.53%)	156676 (99.54%)
Mean Read Length	153	159	136	146	137
Completeness	94.69%	94.75%	94.69%	94.82%	94.75
GC content (%)	38.51%	38.17%	38.04%	37.80%	39.10%
Expected variants	**RT**: V179I **PRO**: K20I, M36I, L63S, H69K, L89M	**RT**: A98G, K103N, M184V, T215Y, M230L **PRO**: M36I, L63A, H69K, I93L	**RT**: K103KQ **PRO**: I64V, V77I, I93L	**RT**: - **PRO**: M36I, D60E, I64V	**RT**: D67N, K70R, M184V, K219Q **PRO**: M36I, H69K, L89M, I93L
Unexpected variants	-	-	-	**PRO**: A71AT	-

**Table 4:** Assessment of Vela NGS platform performances on EQA samples—INT Regions.

INT	Sample 1	Sample 2	Sample 3	Sample 4	Sample 5
Subtype	CRF_02AG	C	B	D	C
Median coverage	3597	9127	8057	8038	7665
Total Mapped Reads	70,284 (99.23%)	180,631 (99.54%)	157,328 (99.69%)	164,132 (99.73%)	143,912 (99.5%)
Read Length	159	153	158	139	144
Completeness	98.64%	98.64%	98.64	98.64%	98.64%
GC content (%)	38.61%	38.69%	38.05%	37.80%	38.95%
Expected variants	T125A, G163E	T125P	-	T125TA	T125A
Unexpected variants	-	-	T125TM	T125A	-

**Table 5:** Assessment of Sentosa^®^ SQ HIV Genotyping Assay performance on intra-assay reproducibility.

Sample	Viral Load (cp/mL)	Subtype	Mapped Reads	Coverage	No. Mismatches
P. 1_A	928,859	B	158218 (99.46%)	PRO/RT: 7543 INT: 7743	0
P. 1_B			130509 (99.46%)	PRO/RT: 9511 INT: 3640	
P. 2_A	66,857	B	69321 (99.48%)	PRO/RT: 4956 INT: 2629	2
P. 2_B			38263 (99.48%)	PRO/RT: 3165 ^a^ INT: 1570	
P. 3_A	32,500	G	117138 (99.29%)	PRO/RT: 9553 INT: 4119	0
P. 3_B			174204 (99.38%)	PRO/RT: 13,870 INT: 6268	
P. 4_A	14,900	B	121373 (99.47%)	PRO/RT: 8116 INT: 3746	0
P. 4_B			111858 (99.0%)	PRO/RT: 7262 INT: 3689	
P. 5_A	737	A1	33565 (98.92%)	PRO/RT: 106 ^b^ INT: 1697	1
P. 5_B			36386 (99.2%)	PRO/RT: 107 INT: 1774	
P. 6_A	29,512	C	104346 (99.34%)	PRO/RT: 6518 INT: 5266	0
P. 6_B			77317 (99.61%)	PRO/RT: 4658 INT: 4034	
P. 7_A	37,153	C	41770 (99.45%)	PRO/RT: 2920 INT: 2188	0
P. 7_B			29267 (99.34%)	PRO/RT: 2340 INT: 966	
P. 8_A	112,202	C	64745 (99.66%)	PRO/RT: 4001 INT: 3925	0
P. 8_B			68129 (99.43%)	PRO/RT: 5364 INT: 3591	
P. 9_A	144,544	B	132430 (99.59%)	PRO/RT: 10,017 INT: 6036	0
P. 9_B			79885 (99.75%)	PRO/RT: 4584 INT: 5027	

^a^ PRO codons 1–61, RT codons 208–386. ^b^ PRO codons 61–99, RT codons 1–324.

**Table 6:** Assessment of Sentosa^®^ SQ HIV Genotyping Assay performance on inter-assay reproducibility.

Sample	Viral Load (cp/mL)	Subtype	Mapped Reads	Coverage	No. Mismatches
P. 6_Run A	423,608	G	214,315 (99.23%)	PRO/RT: 15,033 INT: 8023	0
P. 6_Run B			215,497 (99.39%)	PRO/RT: 15,416 INT: 8280	
* P. 7_Run A	173,785	B	19,908 (98.29%)	PRO/RT: 910 INT: 901	0
* P. 7_Run B			20,764 (98.68%)	PRO/RT: 970 INT: 925	
P. 8_Run A	149,917	B	138,132 (99.41%)	PRO/RT: 8734 INT: 8051	0
P. 8_Run B			141,556 (99.54%)	PRO/RT: 9072 INT: 8567	
P. 9_Run A	129,202	A1	62,630 (98.69%)	PRO/RT: 3995 INT: 3091	0
P. 9_Run B			63,129 (98.87%)	PRO/RT: 4025 INT: 3145	
P. 10_Run A	63,341	B	161,624 (99.06%)	PRO/RT: 9508 INT: 8339	1
P. 10_Run B			160,832 (99.19%)	PRO/RT: 9567 INT: 8635	
* P. 11_Run A	56,746	B	1974 (96.67%)	PRO/RT: 121 ^a^ INT: 58	0
* P. 11_Run B			50,592 (99.37%)	PRO/RT: 1866 INT: 4057	
* P. 12_Run A	25,387	C	8102 (98.55%)	PRO/RT: 623 INT: 304	0
* P. 12_Run B			8053 (99.0%)	PRO/RT: 649 INT: 319	
P. 13_Run A	6590	B	135,416 (99.49%)	PRO/RT: 7207 INT: 6910	0
P. 13_Run B			134,018 (99.58%)	PRO/RT: 7136 INT: 6963	
* P. 14_Run A	5953	A1	9707 (99.18%)	PRO/RT: 713 INT: 267	3
* P. 14_Run B			9193 (99.34%)	PRO/RT: 712 INT: 259	
* P. 15_Run A	936	B	13,183 (99.07%)	PRO/RT: 1093 INT: 302	0
* P. 15_Run B			13,279 (99.25%)	PRO/RT: 1091 INT: 326	
* P. 16_Run A	922	CRF_02AG	2013 (97.62%)	PRO/RT: 85 ^b^ INT: 25	1
* P. 16_Run B			2004 (98.09%)	PRO/RT: 89 ^c^ INT: 28	
* P. 17_Run A	489	A1	5583 (98.24%)	PRO/RT: 176 INT: 230	0
* P. 17_Run B			5384 (98.81%)	PRO/RT: 156 INT: 224	
P. 18_Run A	480	B	136,968 (99.2%)	PRO/RT: 4129 INT: 4848	0
P. 18_Run B			135,374 (99.35%)	PRO/RT: 4105 INT: 4854	
P. 19_Run A	319	B	45,798 (99.78%)	PRO/RT: 1936 INT: NA	NA
P. 19_Run B			19,752 (99.26%)	PRO/RT: NA INT: 339	

^a^ PRO cod. 24–99. ^b^ PRO cod. 64–99. No INT seq. ^c^ PRO cod. 41–99. INT cod. 251–261 * Warning message due to low throughput.

## Data Availability

The data presented in this study are available on reasonable request from the corresponding author.

## Supplementary Materials

The following are available online at https://www.mdpi.com/article/10.3390/ijms23052727/s1.
