# Supplementary material for: Analytical Assessment of the Vela Diagnostics NGS Assay for HIV Genotyping and Resistance Testing: The Apulian Experience
Source: Int J Mol Sci. 2022 Mar 1;23(5):2727. doi: 10.3390/ijms23052727 (PMC8911269; doi:10.3390/ijms23052727)
Supplement: Supplementary file 1 [file ijms-23-02727-s001.zip › ijms-1575986-supplementary.pdf]

Supplementary Material

Analytical Assessment of the Vela Diagnostics NGS Assay for HIV Genotyping and Resistance Testing: The Apulian Experience

Maria Addolorata Bonifacio [1], Chiara Genchi [1], Antonella Lagioia [2], Vincenza Talamo [1] , Anna Volpe [2,*] and Maria Addolorata Mariggiò [1*]

[1] Section of Experimental and Clinical Pathology, Department of Biomedical Sciences and Human Oncology, University of Bari Aldo Moro, Bari, Italy; maria.bonifacio@uniba.it; chiaragenchi.policlinico@gmail.com; mariaaddolorata.mariggio@uniba.it; vincenzatalamo.policlinico@gmail.com

[2] Clinic of Infectious Diseases, Department of Biomedical Sciences and Human Oncology, University of Bari Aldo Moro, Bari, Italy; antonella.lagioia@uniba.it; anna.volpe@uniba.it

* Correspondence: anna.volpe@uniba.it; Tel.: +39 0805592185/3383815430

Supplementary material reported below:

Table S1. Variants and RAMs called by HIVdb program v. 9.0 (2021-02-22), not detected by Sentosa® SQ Reporter server. The underlined RAM was not reported by Sentosa® SQ Reporter server.

Table S2. Variants detected by Sentosa® NGS platform within the inter-assay reproducibility test and their relevant frequencies. RAMs (n=8) are reported in blue. Underlined variants (n=5) do not match between the two runs.

Table S1. Variants and RAMs called by HIVdb program v. 9.0 (2021-02-22), not detected by Sentosa® SQ Reporter server. The underlined RAM was not reported by Sentosa® SQ Reporter server.

Sample	Variants detected by HIVdb v. 9.0, not called by Sentosa® SQ Reporter
P. 1	**PRO:** I13V, I15IV, L63T, E65D<br>**RT:** E53ED, V60VI, K122E, D123S, I135T, K173R, D177E, R211K, V245E, A272P, K277R, I293V, E297K, D324E, I326IT, R358K, K366R, A376V, S379SCG<br>**INT:** S17N, L28I, S57N, K111R, T122TI, T124N, T125A, V201VI, T206S, D232DE, D253E, A265AV
P. 2	**PRO:** N37S, R41K<br>**RT:** V35I, R83K, K103KE, D123DE, I135IV, T165TI, K166KT, Q174QK, G196E, V245K, A272P, K281R, A288AT, E297K, V317A, Y319YH, I326IR, F346Y, S379SCG<br>**INT:** S17N, I72V, P90P*KQST, A91AT, I113IV, T124N, T125V, M154L, V165I, A179AT, V201I, Q216H, D256E
P. 3	**PRO:** I13V, K14R, I50IN, I15IV, E35Q, N37D, R41K, R57K, C67E, H69R<br>**RT:** V35T, E40D, V60I, K102Q, K122E, D123S, K173T, Q174K, T200A, Q207D, R211RK, V245Q, D250E, S251D, A272P, T286TA, E291D, V292I, I293V, P294T, E297AG, D324E, I329V, Q334H, G335D, F346Y, R356K, M357R, R358G, G359PS, V365I, K366R, A376S, S379SCG<br>**INT:** F1FV, E11ED, V31I, S39SG, K42KR, L74I, L101I, T112IMV, G134N, K136T, I162V, V201I, T206S, K211R, K219Q, L234I, S255N, D256E, S283G
P. 4	**PRO:** P39PT, L63A<br>**RT:** K122E, D123E, I178M, R211K, V245K, A272P, K277R, K281KR, T286A, V292I, I293IV, E297K, A360AT, S379SCG<br>**INT:** S17SN, H51HQ, I72T, K111KR, T124N, T125IV, M154L, V201VI, K211R, D288DN
P. 5	**PRO:** L10I, I13V, E21K, L33LV, E35D, R41K, L63M<br>**RT:** V8I, V35T, E40D, K49R, V60I, V90VA, I135T, K166T, K173S, D177E, E194D, T200A, Q207A, R211S, V245K, D250E, S251C, K277R, T286A, E291D, V292I, I293V, P294T, K311R, G335D, P345Q, R356RK, M357K, G359S, A360T, K366R, T369A, A371V, I375V, A376V, T377M, S379C<br>**INT:** E10EK, K14R, R20RK, V31I, V32I, D41DN, V54VI, I60IM, L63LI, E69EK, G70GE, I72V, T112V, I113V, T124A, T125A, V126F, G134N, I135V, K136Q, G149GE, D167E, K173R, V201I, K211KR, T218S, L234IV, V259VI, S283G
P. 6	**PRO:** T12S, I15V, L19T, R41K<br>**RT:** V35T, T39E, S48T, V60I, K122E, D123N, T139K, S162Y, K173A, Q174K, D177E, T200A, Q207E, V245K, A272P, K277R, T286A, E291D, V292I, I293V, D324E, Q334D, R356E, G359T, E370A, A376S, T377M, S379SCG<br>**INT:** D25E, V31I, M50I, I72V, F100Y, L101I, T112V, S119T, T124A, T125A, K136Q, K188R, V201I, K211R, T218I, L234I, A265V, R269K, D278A, S283G
P. 7	**PRO:** L19I, E35D, R41K, Q61E<br>**RT:** V35T, T39E, S48T, E53D, V111I, K122E, D123S, K173A, Q174K, D177E, G196E, T200A, E203V, Q207E, H208Y, R211K, V245Q, E248D, A272P, K277R, T286A, A288T, E291D, V292I, I293V, Q334N, G335D, R356K, G359T, T377M, S379SCG<br>**INT:** S17N, V31I, L45Q, I72V, F100Y, L101I, T112V, S119T, T124S, T125A, K136Q, V201I, T206S, T218I, L234I, R269K, D278A, S283G, R284G, D286N
P. 8	**PRO:** L19I, E35D, R41K, Q61E<br>**RT:** V35T, T39E, S48T, E53D, V111I, K122E, D123S, K173A, Q174K, D177E, G196E, T200A, E203V, Q207E, H208Y, R211K, V245Q, E248D, A272P, K277R, T286A, A288T, E291D, V292I, I293V, Q334N, G335D, R356K, G359T, T377M, S379SCG<br>**INT:** S17N, V31I, L45Q, I72V, F100Y, L101I, T112V, S119T, T124S, T125A, K136Q, V201I, T206S, T218I, L234I, R269K, D278A, S283G, R284G, D286N
P. 9	**PRO:** G17GA, Q18QE, N37S<br>**RT:** E6D, K43E, T200I, R211K, K220KR, V245T, E248D, A272P, K277R, A376T, S379SCG<br>**INT:** <u>D232N</u>, E10D, I72V, T124A

Table S2. Variants detected by Sentosa® NGS platform within the inter-assay reproducibility test and their relevant frequencies. RAMs (n=8) are reported in blue. Underlined variants (n=5) do not match between the two runs.

Sample	Detected Mutations and frequency %
P. 6_Run A	**PRO:** K20I (99.51%), M36I (99.83%), I64M (97.68%), H69K (99.3%), V82I (99.89%), L89M (99.85%) **INT:** T97A (99.67%), T206S (99.85%)
P. 6_Run B	**PRO:** K20I (99.54%), M36I (99.74%), I64M (97.93%), H69K (99.13%), V82I (99.94%), L89M (99.88%) **INT:** T97A (99.72%), T206S (99.76%)
*P. 7_Run A	**PRO:** M36I (5.18%), L63P (99.38%) **RT:** A98S (93.14%) **INT:** G193E (98.72%)
*P. 7_Run B	**PRO:** M36I (6.9%), L63P (99.62%) **RT:** A98S (93.35%) **INT:** G193E (99.36%)
P. 8_Run A	**PRO:** I15V (11.52%), G16E (99.12%), I62V (82.28%), I64V (89.9%), V77I (99.83%) **RT:** A98S (97.96%) **INT:** G193E (99.4%)
P. 8_Run B	**PRO:** I15V (11.53%), G16E (99.09%), I62V (83.17%), I64V (90.15%), V77I (99.89%) **RT:** A98S (97.92%) **INT:** G193E (99.45%)
P. 9_Run A	**PRO:** L10I (75.99%), L10V (23.0%), I15V (23.06%), G16E (74.78%), K20R (71.21%), M36I (98.67%), H69K (98.69%), L89M (99.8%) **RT:** V179I (91.89%) **INT:** L74I (97.78%)
P. 9_Run B	**PRO:** L10I (74.0%), L10V (25.27%), I15V (24.31%), G16E (72.65%), K20R (68.04%), M36I (98.41%), H69K (97.89%), L89M (99.49%) **RT:** V179I (92.32%) **INT:** L74I (98.49%)
P. 10_Run A	**PRO:** G16E (98.84%), I62V (99.74%), V77I (99.61%) **RT:** A98S (98.59%) **INT:** G193E (99.36%)
P. 10_Run B	**PRO:** G16E (98.92%), I62V (99.68%), V77I (99.76%) **RT:** A98S (98.76%) **INT:** K156N (3.42%), G193E (99.31%)
*P. 11_Run A	**PRO:** M36I (98.36%), L63P (97.3%), H69K (93.33%)
*P. 11_Run B	**PRO:** M36I (97.03%), L63P (99.75%), H69K (96.78%)
*P. 12_Run A	**PRO:** L10V (98.53%), I15V (100.0%), G16E (93.1%), H69K (94.85%), L89I (98.98%), I93L (99.76%) **RT:** V179I (98.76%)
*P. 12_Run B	**PRO:** L10V (97.06%), I15V (100.0%), G16E (95.7%), H69K (93.65%), L89I (98.82%), I93L (99.47%) **RT:** V179I (100.0%)
P. 13_Run A	**PRO:** I15V (91.66%), L63P (96.06%), V77I (99.84%), I93L (99.75%) **INT:** L74I(99.53%), G163E (51.67%)
P. 13_Run B	**PRO:** I15V (93.01%), L63P (97.06%), V77I (99.85%), I93L (99.71%) **INT:** L74I (99.55%), G163E (49.58%)
*P. 14_Run A	**PRO:** M36I (99.42%), I64L (94.52%), L89M (99.6%) **INT:** L74I (99.3%)
*P. 14_Run B	**PRO:** M36I (99.68%), H69K (87.69%), H69Q (13.25%), L89M (99.78%) **INT:** L74I (99.54%)
*P. 15_Run A	**PRO:** I15V (95.83%), G16E (98.0%), M36I (97.08%), I62V (99.8%), L63P (99.42%), I93L (75.79%) **RT:** K101E (98.55%), E138Q (98.41%), V179I (99.66%), Y181I (98.17%), M184V (99.67%), K219Q (99.56%) **INT:** T206S (99.5%)
*P. 15_Run B	**PRO:** I15V (98.78%), G16E (100.0%), M36I (96.88%), I62V (100.0%), L63P (99.21%), I93L (77.66%) **RT:** K101E (98.66%), E138Q (97.81%), V179I (99.39%), Y181I (97.7%), M184V (99.74%), K219Q (98.94%) **INT:** T206S (99.76%)
*P. 16_Run A	**PRO:** L89M (100.0%)
*P. 16_Run B	**PRO:** H69K (98.25%), L89M (98.51%)
*P. 17_Run A	**PRO:** L33V (100.0%), M36L (100.0%), L63P (99.05%), H69K (97.27%), T74S (97.37%), L89I (99.23%), I93L (99.22%) **INT:** L74I (99.62%), G163E (98.73%)
*P. 17_Run B	**PRO:** L33V (100.0%), M36L (100.0%), L63P (100.0%), H69K (100.0%), T74S (98.96%), L89I (100.0%), I93L (100.0%) **INT:** L74I (99.55%), G163E (98.86%)
P. 18_Run A	**PRO:** V77I (99.88%), I93L (99.84%) **INT:** E138K (99.76%), Q148R (99.79%)
P. 18_Run B	**PRO:** V77I (99.7%), I93L (99.98%) **INT:** E138K (99.83%), Q148R (99.65%)
P. 19_Run A	**PRO:** L63P (99.94%), V77I (99.79%), I93L (99.9%)
P. 19_Run B	**PRO/RT:** No seq.
